# Mean kinetic temperature evaluations through simulated temperature excursions and risk assessment with oral dosage usage for health programs

**DOI:** 10.1186/s12889-022-12660-9

**Published:** 2022-02-14

**Authors:** David Jenkins, Aida Cancel, Thomas Layloff

**Affiliations:** 1 Product Quality and Compliance, FHI 360, 2810 Meridian Parkway, Suite 160, Durham, NC 27713 USA; 2 Retired, formerly with Product Quality and Compliance, FHI 360, 2810 Meridian Parkway, Suite 160, Durham, NC 27713 USA

**Keywords:** Temperature excursion, Mean kinetic temperature, Shelf-life, Risk assessment, Resource management

## Abstract

**Background:**

Temperature excursions occur during the transport and storage of pharmaceuticals, and often result in considerable losses for public health programs operating in countries with limited resources. After a temperature excursion has been identified, often products are discarded without any additional risk assessments. Consulting the manufacturer is the preferred approach but can be challenging depending on the responsiveness of the manufacturer. However, decisions are often required quickly depending on program needs and available stock in country.

**Methods:**

To provide further guidance, simulations have been conducted based on mean kinetic temperature evaluations using accepted default kinetic parameters to assess loss of shelf-life for scenarios involving various levels of temperature excursions on a model pharmaceutical at different recommended storage conditions, shelf-life, and long-term storage conditions.

**Results:**

Although an immediate loss to shelf-life occurred with excursions when the product was stored at the maximum allowed temperature, more extended excursion could be withstood before loss of shelf-life was detected when long-term storage was maintained at temperatures below the maximum storage condition for the product. With the assumption that a shelf-life loss of 2 weeks was negligible when managing program stock, a risk assessment was conducted to outline the various times that excursions at different temperatures could be considered low risk to the program.

**Conclusions:**

Depending on the level of the temperature excursion and the guidance provided by the manufacturer, public health programs will have further information with this assessment to guide decisions that impact safety to the end user and resource management due to temperature excursions that can occur.

**Supplementary Information:**

The online version contains supplementary material available at 10.1186/s12889-022-12660-9.

## Background

Pharmaceutical products have an expiration date based on stability studies conducted under controlled temperature and humidity conditions which establishes the product’s recommended transport and storage environmental conditions [1, 2]. Pharmaceutical products are to be stored as per the manufacturer’s recommended storage conditions described in the label so that products are of acceptable quality throughout its established shelf-life.

However for public health programs through donor agencies (excluding cold-chain products), pharmaceutical products labeled with the usual storage temperatures of 25 °C or 30 °C [3] are often transported in non-refrigerated containers, potentially passing through different climate zones ranging from very cold to very hot temperatures (commonly found in Africa, portions of Asia and South America). Once in country, the products are purportedly stored under good storage conditions at temperatures at or below the required storage temperature. The product may be transferred thorough distribution channels and supplied in non-temperature regulated vehicles to health centers and hospitals where it may be stored for varying periods before eventually being supplied to the patient. Although guidance is available on good distribution practices [4, 5], the temperature and humidity values reached during storage and transportation routes can vary depending on the route, weather conditions and transport vehicles [6, 7] due to limited ability to monitor and control in limited resource settings. For example, an evaluation of pharmaceutical shipments sent by ocean from UNICEF in Copenhagen, Denmark to (a) Lagos, Nigeria, to (b) Mombasa, Kenya and then by land to Kampala, Uganda, and (c) to Bangkok, Thailand of various medicines stored in multiple locations in transport showed temperatures from − 3.5 °C to 42.4 °C and relative humidity ranges from 20 to 88% [8].

Devices are available to accompany shipments in order to monitor for temperature excursions (period of exposure to temperatures exceeding the designated range for the product [9]) during transport and storage [10, 11]. Although the data from these monitors indicate whether an excursion has occurred, the severity of the excursion and the risk to the product are likely to be less understood and may need to be assessed. Furthermore, the true temperature experienced by the product may be difficult to accurately determine (potentially complicating assessments) depending on the exact placement of the data logger, type of packaging, and the equilibration rate of the product’s temperature relative to its surroundings over brief periods of time (i.e., hours). Loss of product integrity (i.e., loss of potency, increase of degradation products) due to temperature excursions could increase risk or decrease benefit to the patient if used [12]; in contrast subsequent removal of product from the supply chain may lead to stock outs and wastage that present their own risks. Also, significant financial loss is incurred when lots are not recommended for distribution after experiencing a temperature excursion. From a regulatory perspective, the manufacturer is responsible for addressing temperature excursions when these occur. However, the information on the effects of temporary excursions are not always addressed in the labeling information and retrieving information from each manufacturer is complicated by prolonged response times, lack of available data, and the inability of some manufacturers to release the information.

To help procurers address these complications, modeling results for various temperature excursion scenarios that estimate the loss of shelf-life to pharmaceuticals and the potential impact to the supply chain are presented based on a key product quality attribute as assessed by mean kinetic temperature evaluations based on the Arrhenius equation for reaction kinetics. This assessment provides some guidance to public health programs that are required to manage the transport and storage logistics of various life-saving commodities, and that often are required to make risk-based decisions on product usage or removal for large inventories that may have experienced adverse environmental conditions.

### Assessment of product quality attributes to establish the model framework

Important product quality attributes that can be influenced by temperature excursions during transport and storage are potency (loss of assay), dissolution (change in API release profile), impurities (increase in impurities or degradation products), and microbial contamination (discoloration due to microbial growth) [2, 12]. The overall consequence of the occurrence is that changes to these attributes may impact product degradation and microbial growth, which in turn may impact overall product therapeutic effectiveness and shelf-life.

Generally, most pharmaceutical forms used in public health programs are solid dosage forms. These dosages are a mixture of active pharmaceutical ingredients and a variety of other excipients either in a compressed tablet or capsule. The modelling assessment for this work is therefore focused on solid dosages, where the amount of API (assay) is the key parameter used in the calculations. Assay has been focused on for these simulations because the minimum assay specification of 90% through the end of the shelf-life is very common across pharmaceutical products. For other pharmaceutical quality parameters previously mentioned, the range of product specifications was considered more difficult to generalize and was not pursued here. Any one of these parameters may be a key driving force behind a certain product’s shelf-life. Truly knowing this relies on the manufacturer’s expertise, which as stated before may be difficult to obtain under certain circumstances and motivated this assessment. Packaging is assumed not be significantly affected by humidity. Recommended storage conditions for the products is assumed to be either 25 °C or 30 °C (depending on the specific scenario), due to the prominence of these storage conditions in finished products [3] and that WHO prequalified products [13] are observed that adhere to either of these conditions to provide coverage across climatic zones II and IV (a and b) [1, 2].

### Review of mean kinetic temperature and the Arrhenius equation

Temperature excursions can be defined as environmental temperatures to which a product may be exposed during transport or long-term storage that exceed the manufacturer’s label claim conditions for the product. A common storage condition for many pharmaceuticals is “controlled room temperature;” the USP describes “controlled room temperature” as a temperature that includes the typical environment of 20 °C to 25 °C (68 °F to 77 °F), where excursions are allowed between 15 °C and 30 °C (59 °F and 86 °F) provided that the mean kinetic temperature (T_k_) remains at or below 25 °C [14]. Furthermore (provided the T_k_ does not surpass 25 °C), the USP recommends that excursions up to 40 °C occur for no more than 24 h without consultation with the manufacturer [14].

Mean kinetic temperature (T_k_) is a calculated temperature where the level of degradation over a certain time period is the same as the total of separate degradations that could result from a series of different temperatures throughout the same total time period, respectively [15]. When measured temperatures experienced by a sample are available for a series of time periods (i.e., for example with a regular periodicity of hours or days), T_k_ can be calculated using Eq. 1 below [15–17], where E_a_ is the

1 $${T}_k=\frac{E_a/R}{-\mathit{\ln}\left(\frac{e^{\left(-{E}_a/R{T}_1\right)}+{e}^{\left(-{E}_a/R{T}_2\right)}+\dots +{e}^{\left(-{E}_a/R{T}_n\right)}}{n}\right)}$$

activation energy, R is the universal gas constant, n is the number of time points in the overall series, and T_n_ is the absolute temperature (K) for each of the time periods 1 through n, respectively.

The calculation of T_k_ is based on the application of the Arrhenius model for reaction kinetics. Focusing on the degradation of active pharmaceutical ingredient B, the degradation of B can be represented with the generic reaction shown in Eq. 2, and the rate of reaction with respect to reactant B is provided in Eq. 3, where [B] is the reactant concentration, k is the rate constant for the reaction, and

$$\mathrm{B}\to \mathrm{degradation}\ \mathrm{products}$$

3 $$\frac{-d\left[B\right]}{dt}=k{\left[B\right]}^{\beta }$$

β is the reaction order for reactant B [18].

The rate constant (k) is an important parameter to characterize and can be determined by measuring reactant concentrations as a function of time. The rate constant (k) can be modeled with the Arrhenius equation shown in Eq. 4, where A is the frequency factor. Key features of the Arrhenius

4 $$k={Ae}^{-{E}_a/ RT}$$

equation are that k increases exponentially with temperature, where E_a_ and A are modeled as constants [18]. For a given reaction, E_a_ and A can be determined from the slope and intercept, respectively, of the linearized form of the Arrhenius equation, Eq. (5), by plotting experimentally

5 $$\ln \left(\mathrm{k}\right)=\ln \left(\mathrm{A}\right)-\left({\mathrm{E}}_{\mathrm{a}}/\mathrm{RT}\right)$$

determined k’s as a function of absolute temperature. With E_a_ and A, the rate constant (k) can be calculated at a specific temperature and subsequently used to determine the rate of reaction [19]. Basically, the Arrhenius equation demonstrates that a rate of reaction will increase exponentially with temperature, rather than linearly. Correspondingly, the mean kinetic temperature is important to understand because it provides the effective isothermal temperature experienced by the product that accounts for the Arrhenius-based effect of temperature excursions during storage [15].

## Methods

### Modeling impact of temperature excursions on shelf-life

To estimate the impact of temperature excursions on a pharmaceutical product’s shelf-life, various scenarios were evaluated with the different parameters and long-term storage conditions (outside of the excursions) outlined in Table 1.

**Table 1:** Scenarios used for various temperature excursion calculations

Scenario	Parameters	Long term storage conditions employed after excursion
1	25 °C label storage condition with 2-year expiration date. Assume assay reaches 90% in 24 months at 25 °C.	Scenario 1a – 25 °C
		Scenario 1b – 22 °C
2	25 °C label storage condition with 5-year expiration date. Assume assay reaches 90% in 60 months at 25 °C.	Scenario 2a – 25 °C
		Scenario 2b – 22 °C
3	30 °C label storage condition with 2-year expiration date. Assume assay reaches 90% in 24 months at 30 °C.	Scenario 3a – 30 °C
		Scenario 3b – 27 °C
4	30 °C label storage condition with 5-year expiration date. Assume assay reaches 90% in 60 months at 30 °C.	Scenario 4a – 30 °C
		Scenario 4b – 27 °C

To generate all data for this study (see Supplemental File 1), all scenarios were modelled under two main assumptions. The first assumption is that stability data was unknown or unavailable from the product’s manufacturer, which allows the use of 83.144 kJ/mol as a default E_a_ [15, 17]. For the second assumption, kinetic calculations would be conducted based on a zero-order rate law of Eq. 2. (β equals 0 in Eq. 3), where the actual amount of B is based on the percent label claim of the active ingredient B yielding the simplified rate law as summarized in Eqs. 6 and 7.

6 $$\frac{d\left[B\right]}{dt}=-k$$

7 $${\left[B\right]}_0-{\left[B\right]}_t= kt$$

Although many degradations may follow first (or other) orders, the use of zero-order simplifies the calculations and makes minimal difference in the early stages (~ 10% change in reactant level) relative to other orders [19]. The use of reactant percent (i.e., as opposed to a concentration in mol/L) has been implemented in pharmaceutical kinetic evaluations for assay [20], degradation products [21], and dissolution profiles [22]. These calculations will focus on the change in assay, where the active ingredient amount is 100% label claim (LC) initially and is assumed to reach 90% at the end of the shelf-life for time t [20], yielding a further simplification of Eq. 7 to the following in Eq. 8.

8 $${\left[100\% LC\right]}_0-{\left[90\% LC\right]}_t= kt$$

With Eq. 8, the rate constant (k) for each scenario was calculated (yielding the k at 25 °C for scenarios 1 and 2 and the k at 30 °C for scenarios 3 and 4) and then used with Eq. 4 to determine the frequency factors (A) for each scenario, respectively (see Table 2).

**Table 2:** Kinetic parameters incorporated for the different temperature excursion calculations based on a zero-order assumption, 30-day month, and default activation energy (E_a_) of 83.144 kJ/mol [2, 4]

Scenario	% Label Claim (LC) - Assay	Days (through shelf life)	k (% LC / day)	A (%LC / day)
1	100	0	0.01389	5.15371 × 10^12^
	90	720		
2	100	0	0.00556	2.06148 × 10^12^
	90	1800		
3	100	0	0.01389	2.96332 × 10^12^
	90	720		
4	100	0	0.00556	1.18533 × 10^12^
	90	1800		

Mean kinetic temperature calculation templates (Supplemental File 1) were generated in Microsoft® Office Excel to calculate 720-day (2 year) simulations for scenarios 1 and 3 and 1800-day (5 year) simulations for scenarios 2 and 4, respectively. The template was established to operate over a frequency of days to estimate the mean kinetic temperature over the entire 2- or 5-year period for these simulations, respectively. In reality, data loggers can provide readings over frequencies of hours or 12-h intervals (for example). For an actual field excursion with data logger readings, the calculations described here could be readily repeated to accommodate the exact field situation. For this set of simulations, each template’s T(°C) column was populated for each day with the following values (respectively for the listed scenarios): 25 °C (1a, 2a); 22 °C (1b, 2b); 30 °C (3a, 4a); 27 °C (3b, 4b). Using the templates, products with 25 °C storage conditions (scenarios 1 and 2) were evaluated for a series of full day excursions at 30 °C, 40 °C, 50 °C, and 60 °C (where the product is assumed to be equilibrated to these temperatures), each ranging from 1 day to multiple consecutive days of excursions (each series increasing incrementally by full day time periods) to ultimately calculate when at least 20–30 days of shelf-life was lost. In the same manner, products with 30 °C storage conditions (scenarios 3 and 4) were evaluated for temperature excursion of 40 °C, 50 °C, and 60 °C.

For each temperature excursion series (per scenario), the mean kinetic temperature was calculated respectively for multiple excursion periods (1 day, 2 days, 3 days, etc.). For each mean kinetic temperature calculation, the remaining shelf-life for the product (t) was estimated based on Eq. 9, and adjusted specifically to Eq. 10, where t is the time in days required to reach an assay of 90% LC, A is the

9 $$t=\frac{{\left[100\% LC\right]}_o-{\left[90\% LC\right]}_t}{k}$$

10 $$t=\frac{10}{\left({Ae}^{-{E}_a/ RT}\right)}$$

frequency factor for specific for each scenario from Table 2, E_a_ is the default activation energy of 83.144 kJ/mol, R is the universal constant (0.0083144 kJ/mol K), and T is the specific mean kinetic temperature in absolute Kelvin calculated for each excursion period. This calculation was repeated for all scenarios to estimate the impact on shelf-life loss resulting from various periods of excursions (measured in days) at different temperatures.

## Results

**Fig. 1:**
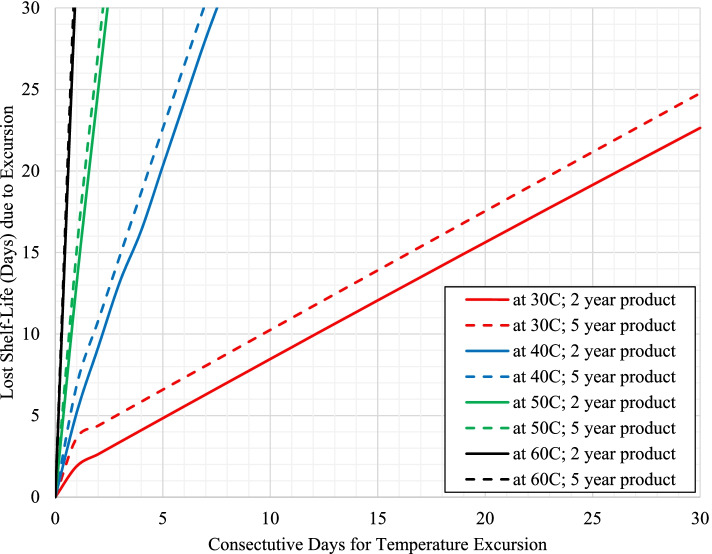
Impact on estimated shelf-life loss from temperature excursions (30 °C, 40 °C, 50 °C, and 60 °C) for product with 25 °C label storage conditions with long term storage at 25 °C (scenarios 1a and 2a)

**Fig. 2:**
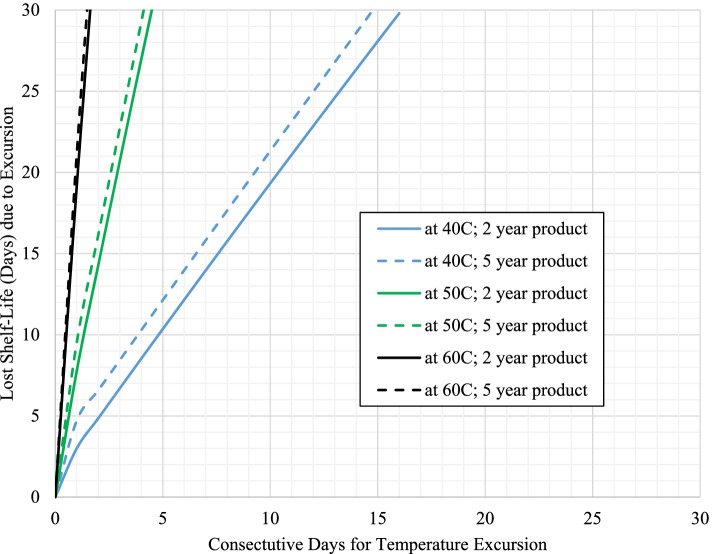
Impact on estimated shelf-life loss from temperature excursions (40 °C, 50 °C, and 60 °C) for product with 30 °C label storage conditions with long term storage at 30 °C (scenarios 3a and 4a)

Temperature excursions have an immediate impact on loss of shelf-life for product with long term storage at temperatures equaling the maximum recommended storage condition as shown in Figs. 1 and 2 for product with 25 °C and 30 °C storage conditions, respectively.

**Fig. 3:**
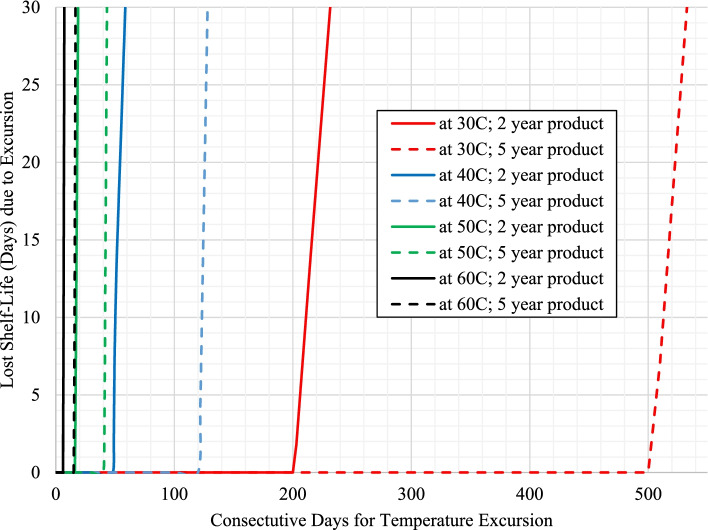
Impact on estimated shelf-life loss from temperature excursions (30 °C, 40 °C, 50 °C, and 60 °C) for product with 25 °C label storage conditions with long term storage at 22 °C (scenarios 1b and 2b)

**Fig. 4:**
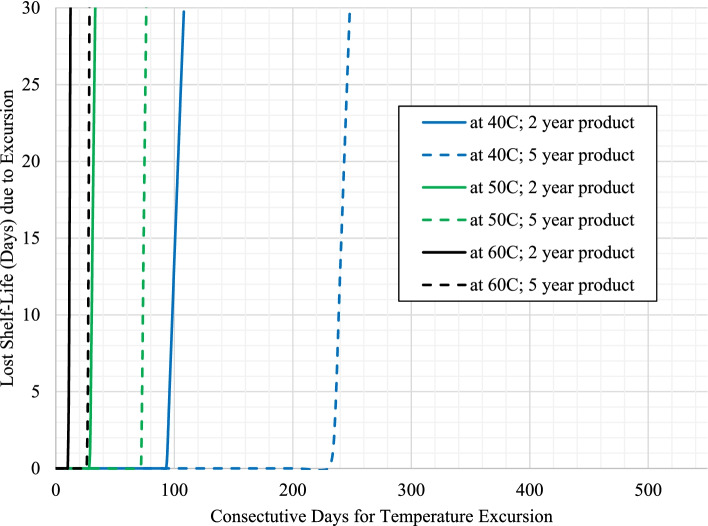
Impact on estimated shelf-life loss from temperature excursions (40 °C, 50 °C, and 60 °C) for product with 30 °C label storage conditions with long term storage at 27 °C (scenarios 3b and 4b)

For these cases, the excursion promptly increases the mean kinetic temperature above the maximum recommended storage temperature for the product resulting in an even further accelerated rate of API loss, where the level increases at higher excursion temperatures and/or with longer excursion times. However, long term storage below the maximum storage temperature for the product (such as 3 °C below) can dramatically delay loss of shelf-life with temperature excursions for both products with either 25 °C or 30 °C storage conditions as shown in Figs. 3 and 4, respectively.

For these cases, the lower long-term storage temperatures allows for longer temperature excursions to occur before the mean kinetic temperature reaches the maximum storage temperature for the product (horizontal portion of the curve), where eventually the longer temperature excursions begin to decrease the estimated shelf-life (observed in the increasing sloped portion of the curve).

To compare the rates of shelf-life loss, Table 3 provides the values for each of the positive slopes from the plots in Figs. 1, 2, 3 and 4 as designated for each scenario / temperature excursion combination.

**Table 3:** Days of shelf-life lost per day of excursion as a function of excursion temperature for each scenario (taken from the region of the curves for each temperature excursion with a positive slope within each respective figure)

**Label Claim Storage / Shelf-life / Long Term Storage**	**30 °C**	**40 °C**	**50 °C**	**60 °C**
Scenario 1a (Fig. 1) – 25 °C / 2 years / 25 °C	0.72	3.77	11.71	28.66
Scenario 2a (Fig. 1) - 25 °C / 5 years / 25 °C	0.73	3.72	11.50	27.26
Scenario 1b (Fig. 3) - 25 °C / 2 years / 22 °C	0.98	2.22	11.99	29.70
Scenario 2b (Fig. 3) - 25 °C / 5 years / 22 °C	1.00	4.18	12.41	32.17
**Average (± 1 SD) for product with 25 °C storage condition**	**0.86 (±0.16)**	**3.48 (±0.86)**	**11.90 (±0.39)**	**29.45 (±2.07)**
Scenario 3a (Fig. 2) - 30 °C / 2 years / 30 °C	n/a	1.79	6.34	17.14
Scenario 4a (Fig. 2) - 30 °C / 5 years / 30 °C	n/a	1.83	6.53	17.75
Scenario 3b (Fig. 4) - 30 °C / 2 years / 27 °C	n/a	2.06	6.65	17.62
Scenario 4b (Fig. 4) - 30 °C / 5 years / 27 °C	n/a	2.10	6.30	18.16
**Average (± 1 SD) for product with 30 °C storage condition**	**n/a**	**1.94 (±0.16)**	**6.45 (±0.16)**	**17.67 (±0.42)**

The rate of shelf-life loss increases within each scenario as excursion temperatures increase. Regardless of the long-term storage condition, products with the same recommended maximum storage condition have similar rates of shelf-life loss (based on the average results shown for each temperature excursion in Table 3), where the rate of shelf-life loss is lower for the product with the 30 °C maximum storage condition. The general consistency observed for each respective product (for either 25 °C or 30 °C storage condition) is apparently because the same default activation energy is used in the calculations, resulting in similar mean kinetic temperature changes for the respective excursions.

The results previously discussed estimate the continual loss of shelf-life due to temperature excursions of various lengths of time. The overall goal of this work was not only to understand the impact on shelf-life, but to also provide guidance on managing the use of product in public health programs that may have experienced a temperature excursion. As a point of reference for managing product inventory, the last 2 weeks remaining in the product’s shelf-life may potentially be considered more expendable relative to the previous longer portion of usable shelf-life for the product. If existing inventory is approaching this minimal amount of remaining use, it is probably in the program’s best interest to designate this product for subsequent disposal. Unless the product can be transported for immediate use, the program may be at risk for dispensing product past the expiration date. Considering this time frame, Table 4 specifically provides the number of consecutive days of temperature excursion that result in a loss of approximately 15 days of shelf-life for each respective scenario.

**Table 4:** Consecutive days of temperature excursion that result in a loss of approximately 15 days of shelf-life for each respective scenario

Label Claim Storage / Shelf-life / Long Term Storage	30 °C	40 °C	50 °C	60 °C
Scenario 1a (Fig. 1) – 25 °C / 2 years / 25 °C	19	3	1	<1
Scenario 2a (Fig. 1) - 25 °C / 5 years / 25 °C	16	3	<1	<1
Scenario 1b (Fig. 3) - 25 °C / 2 years / 22 °C	215	49	17	6
Scenario 2b (Fig. 3) - 25 °C / 5 years / 22 °C	510	124	41	16
Scenario 3a (Fig. 2) - 30 °C / 2 years / 30 °C	n/a	7	2	< 1
Scenario 4a (Fig. 2) - 30 °C / 5 years / 30 °C	n/a	6	1	<1
Scenario 3b (Fig. 4) - 30 °C / 2 years / 27 °C	n/a	100	30	11
Scenario 4b (Fig. 4) - 30 °C / 5 years / 27 °C	n/a	240	74	27

**Fig. 5:**
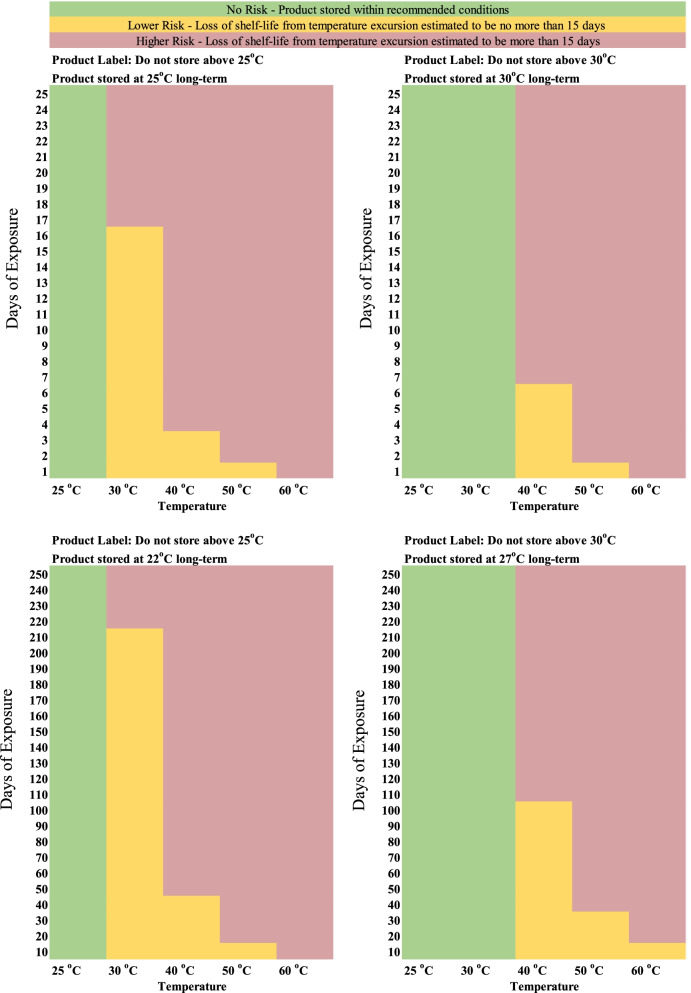
General risk assessment recommendation for product distribution after temperature excursions, assuming a 15-day shelf-life loss is negligible

As these results are directly taken from Figs. 1, 2, 3 and 4, the general trends observed are the same as a function of the magnitude of temperature of excursion, recommended maximum storage temperature, and long-term storage temperature, and have been used to further develop a risk assessment (shown in Fig. 5) for managing product inventory that experiences excursions in environmental temperature. Fig. 5 assumes an approximate two-week loss of shelf-life could be considered negligible.

The risk could be considered low if a temperature excursion outside of the recommended storage condition occurs for time periods below those indicated in Table 4 respectively, where increases in risk are associated with excursions exceeding those timeframes. Although the risks depicted in Fig. 5 indicate a low risk for excursion at 50 °C and 60 °C (based on the calculations), it is highly recommended to consult the manufacturer or other experts highly knowledgeable of the product’s stability profile.

## Discussion

When products experience temperature excursions, the risk adverse approach from the end-user perspective is to remove the product from the supply chain. Although the modeling has been focused on pharmaceuticals, temperature excursion also can occur for medical equipment, vaccines, veterinary medicines, and diagnostics [23–26], where risk mitigation efforts and assessments employed can also apply to pharmaceuticals. To further mitigate risk to the end user, programs may consider using cold chain approaches (commonly applied to vaccines) [27, 28] or explore other temperature-controlled transportation options [29], if the commodity is extremely temperature sensitive. Unfortunately, the implementation of such measures can be financially and logistically burdensome on programs and need to be implemented strategically based on the stability needs for key products. Although efforts to minimize the likelihood of excursions should always be pursued, a balanced approach for evaluating how to proceed in the face of the inevitable occurrence of excursions is needed, and the approach should be based on risk to the end-user, program, and the product.

While investigating the impact of a temperature excursion, information from the manufacturer (especially for excursions up to 40 °C [14]) and their knowledge of the product based on stability data [30, 31] or other types of transportation studies [32], where modeling to determine the specific activation energy for the product is conducted [33] will be critical. Although this may be the best option for developing a mitigation strategy, it may not always readily be feasible depending on the data available or the responsiveness of the manufacturer. This likely information gap provided the motivation for this work. The risk assessment from Fig. 5 assumes that long term storage at slightly cooler temperatures may not be realistic and has been focused on the time points for long term storage at the maximum recommended temperature. If long term storage is possible at slightly cooler temperatures, the risk assessment could be adjusted because long temperature excursions could be tolerated. It should be noted that the simulations for scenarios 1b, 2b, 3b, and 4b, including the estimations indicated in Table 4 and Fig. 5, are based on an assumed 3 °C lower long-term storage relative to the maximum storage temperature for the product to illustrate the impact that lower long-term storage can have on the level of an excursion a product could withstand. Depending on the reality of what a field program’s storage conditions can actually provide, Table 4 and Fig. 5 would need to be adjusted accordingly for scenarios 1b, 2b, 3b, and 4b to the local conditions.

Adherence to manufacturer shelf-life and storage conditions should always be the continual mode of operation. Although product disposal may be the default decision if temperature excursions have occurred, support for product usage after excursions exists under the certain circumstances. Oxytocin ampoules were found to maintain compliance to specifications despite excursions ranging from − 9.9 °C to 30.1 °C [34]. Reviews of epinephrine stability studies found no degradation with cold temperatures and reasonable tolerance to elevated temperatures [35]. Retrospective studies of over 400 cases reporting use of a variety of vaccines after temperature excursions found no substantial health risks, with minimal occurrence of vaccine failure [36]. Monovalent hepatitis B vaccine was found to be tolerant for administration with storage exceeding cold chain conditions [37]. Hogerzeil et al. observed minimal change in API levels for most of the medicines included in a study where product experienced tropical conditions during shipment far exceeding those normally recommended for long term storage [8]. From example data presented over a 14-week period, temperatures ranged from approximately -2 °C to 41 °C during shipment. From this same data, an MKT of 24.4 °C has been calculated by estimating temperatures at the 12-h intervals through the entire period. This study [8] provides evidence that dramatic temperature excursions can occur but may not always result in the need to discard product upon further investigation.

Although continual improvement in management and monitoring can be done to mitigate the likelihood of the occurrence [38, 39], temperature excursions will happen when managing the transport of product across international borders in tropical environments. Testing product for compliance to specifications allows a quality check for that instance in time but does not indicate how much shelf-life remains after the excursion. Even if a program does not have ready access to manufacturer expertise (i.e., from limited data or lack of responsiveness), decisions still need to be made on whether the excursion has made no impact on the product’s shelf-life, has shortened the shelf-life to a level that the product is safe to use but needs to be used more quickly, or the product should be discarded as a result of the excursion. The estimations conducted in this study help to provide a risk assessment to make decisions on product disposition in the event of a temperature excursion when limited information is available on exact nature of the excursion or on the product. The following recommendations may assist health programs in risk mitigation activities after solid-oral dose temperature excursion evaluations:

- Storage facilities (transfer warehouse, logistic warehouse) for pharmaceuticals shall meet at minimum WHO Good Storage Practice requirements [3, 4]. Whenever possible, pharmaceuticals shall be stored below the maximum temperature allowed. Programs may explore maintaining the temperature of the Regional Distribution Centers (RDCs) at temperatures lower than the maximum recommended condition.
- In instances where product distribution after temperature excursion is recommended pharmaceuticals may be tested for critical attributes. The program may also recommend that whenever possible these shall be distributed as soon as possible to complete consumption relatively earlier than the expiration date.
- Temperature excursions above 40 °C are considered extreme temperature excursions and require prioritized program and/or manufacturer quality assurance review (ideally).
- Programs shall include temperature excursions as part of the transport and storage supplier performance data. Collect and periodically evaluate transport and storage supplier performance data.

## Conclusions

When a product experiences a temperature excursion, the manufacturer should be consulted for guidance. For public health programs in resource limited countries, there are situations when access to stability data is challenging, responses from the manufacturer are limited, and decisions need to be made on whether to use pharmaceuticals that have experienced temperature excursions. To provide further guidance, simulations have been conducted on a model pharmaceutical to determine the loss of shelf-life that can occur with different levels of temperature excursions. Immediate loss in shelf-life was experienced when long-term storage matched the maximum storage conditions listed for the product, but more extensive excursions can be experienced before loss to shelf-life occurred when long term storage was below the listed conditions. With an assumed negligible loss in shelf-life of 2 weeks and long-term storage at the allowed temperature maximum, it was considered lower risk for product with a storage temperature of up to 25 °C to experience excursions at 30 °C, 40 °C, and 50 °C for no more than 16–19 days, 3 days, and 1 day, respectively. For product with a maximum storage temperature of 30 °C, low risk was determined for excursions at 40 °C and 50 °C of 6 days and 1 day, respectively. When the product is stored below the recommended label conditions for a long-term after experiencing a temperature excursion, it is possible to extend the product use (i.e., lower risk category) for a longer period relative to a product experiencing an excursion with long term storage at the product’s maximum storage condition, regardless of the storage conditions listed in the label.

## Supplementary Information

**Additional file 1.**

## Data Availability

All data generated or analysed during this study are included in this published article [and its supplementary information files – Supplemental File 1].

## Abbreviations

API: Active Pharmaceutical Ingredient
ICH: The International Council for Harmonisation of Technical Requirements for Pharmaceuticals for Human Use
LC: Label Claim
MKT: Mean Kinetic Temperature
RDC: Region Distribution Center
UNICEF: United Nations International Children’s Emergency Fund
USAID: United States Agency for International Development
USP: United States Pharmacopeia
WHO: World Health Organization
